# One IV HEDGES DNA vector administration encoding hGLA or hGH produces durable hGLA and hGH serum lvels in immunocompetent mice

**DOI:** 10.1371/journal.pone.0318977

**Published:** 2025-10-31

**Authors:** Alice Ye, Marissa Mack, Sarah Ursu, Stephen Chmura, Robert Steiner, Tim Heath, Chakkrapong Handumrongkul, Robert Debs

**Affiliations:** 1 DNARx, San Francisco, CA, USA; 2 University of Wisconsin, Madison, Wisconsin, United States of America; PLOS: Public Library of Science, UNITED STATES OF AMERICA

## Abstract

The great majority of human monogenic, single protein deficiency disease patients, who comprise ~ 0.5% of the population, are incurable. When available, Enzyme Replacement Therapy (ERT) is current state-of-the art therapy for the vast majority of the subset of these diseases caused by enzyme deficiencies. For example, Fabry disease, caused by hemizygous- or heterozygous-pathogenic variants in GLA encoding human-galactocersbrosidase-α (hGLA), is a rare, single protein-deficiency disease. Fabry patients require intravenous-administration of hGLA Enzyme Replacement Therapy (ERT) every two-weeks for-life. ERT costs ~ $300,000 per-year and can cause frequent infusion reactions, which can be life-threatening. The very-high yearly GLA ERT costs, as well as the recurrent, life-threatening hGLA IV infusion-reactions experienced by some patients, can cause them to permanently-discontinue ERT. This can accelerate Fabry-disease progression, leading to premature-death. Therefore, new, more effective-, safe-, durable-, cost-effective, single deficient-protein replacement platforms are urgently-needed to more-effectively treat a wide-spectrum of these rare, monogenic single protein deficiency diseases. Here we demonstrate that one intravenous-administration of our 1^st^-generation HEDGES DNA-vector encoding wildtype-hGLA (T^1^/_2_ < 20-minutes) produced hGLA serum-protein levels in the normal human 1,000-10,000 pg/ml range for only < 14 days. We then-created our 2^nd^-generation HEDGES hGLA DNA-vector. One intravenous-administration of this DNA-vector produced durable (>550 days) serum hGLA serum levels in the 1–10 ng/ml, thus increasing the duration of hGLA serum-protein levels produced by > 38,100 fold versus administering bioreactor-produced, wildtype hGLA-protein. We also showed one intravenous-administration of our 1^st^-generation HEDGES DNA-vector encoding the wildtype human growth hormone (hGH) protein, (T^1^/_2_ < 20-minutes), produced serum hGH levels in the 1–10 ng/ml for > 330 days, thus increasing the duration of hGLA serum-protein levels by > 22,860 fold versus administering wildtype hGH-protein. Last, one intravenous-administration of our 2^nd^-generation HEDGES hGLA DNA-vector produced serum hGLA levels in the normal human 1–10 ng/ml range for > 160 days in GLA knockout-mice, a 2,800-fold increase versus wildtype hGLA-protein. hGLA-ERT produces major therapeutic-responses in GLA knockout-mice. These substantial ERT-responses in GLA knockout-mice have been shown to be accurately-recapitulated in Fabry patients. Thus, Fabry disease appears a promising-target for subsequent phase-1 HEDGES-based human clinical trials.

## Introduction

The great majority of human single protein deficiency diseasees, they comprise ~ 0.5% of the population, remain incurable [1]. When available, ERT is current state-of-the art therapy for the subset of these diseases caused by enzyme deficiencies [2]. However, ERT is almost never curative, requires frequent redosing, usually daily to once every two weeks for life, is inordinately expensive (from $40,000– > $500,000 per-year per patient) as well as can cause life-threatening allergic infusion reactions [3–9]. AAV gene therapy has now revolutionized the treatment for several up to now untreatable rare human monogenic deficiency diseases [10]. However, AAV elicits potentially lethal adaptive immune responses [11], cannot be effectively redosed [12], and can cause insertional mutagenesis, rarely leading to oncogenesis [13].

Fabry disease (FD) is a lysosomal storage disorder caused by pathogenic variants in the *GLA* gene. These variants lead to reduced GLA enzymatic activity, preventing the breakdown of glycolipids including globotriaosylceramide (Gb3) and deacylated globotriaosylsphingosine (lyso-GL-3). Clinically, FD damages the heart, kidneys, nervous system, gastrointestinal tract, and eyes among other tissues [3,4,14–16]. Wild type hGLA protein, like human growth hormone (hGH) protein, has a short protein T^1^/_2_ of < 20 minutes [5,8]. The normal therapeutic range of wildtype hGLA protein is from 1 to 10 ng per ml [5].

The current treatment for FD is recombinant bioreactor produced hGLA ERT which requires bi-weekly IV hGLA protein infusions for the life of the patient. hGLA ERT costs ~ $300,000 per patient per year and is not effective in all patients. The very high yearly hGLA ERT treatment costs, together with recurrent, potentially life-threatening hGLA IV infusion-related toxicities in some patients cause significant numbers of these patients to permanently discontinue ERT, thereby accelerating disease progression which can lead to premature-death [3, 4].

We previously developed HEDGES as a nonviral, intravenous (IV), sequentially-administered, (cationic plus neutral liposomes followed two minutes later by a HEDGES DNA-vector) gene therapy platform. We focused on creating a nonviral gene therapy platform that durably as well as safely produce critical therapeutic effects of a variety of different human proteins, including cytokines as well as monoclonal antibodies (mAbs). Specifically, we showed that one safe, IV administration of our 1^st^-generation HEDGES DNA vector encoding the wild type human G-CSF (hG-CSF) protein (T^1^/_2_ ~ 2 hours) into immunocompetent CD-1 mice produced significantly elevated circulating absolute neutrophil counts (ANC) together with replacement of normal hematopoietic precursor cells with immature neutrophil precursor cells in bone marrow and spleen for > 582 days [17]. The elevation of ANC together with replacement of normal hematopoietic precursor cells with immature neutrophils in bone marrow and spleen is the classic therapeutic response to recombinant hG-CSF protein therapy. Chronic hG-CSF protein replacement therapy is now administered on a daily basis to treat a spectrum of human chronic neutropenic diseases [18]. HEDGES-based hG-CSF administration increased the duration of hG-CSF serum protein levels produced by > 13,900 fold versus recombinant wildtype hG-CSF protein therapy [17].

As hG-CSF, our 1^st^-generation HEDGES platform greatly extended therapeutic activities of FDA-approved mepolizumab and rituximab as well as the 5J8 anti-pandemic influenza mAb. The HEDGES significant longer expression is part due to reduced innate immune responses when compared to either conventional lipoplexes or nanoplexes [17]. A substantial reduction of innate immune responses by HEDGES is accomplished by multiple concurrent modifications to our liposome carriers and nonviral DNA-vector plasmids. This combination of modifications includes: sequential intravenous injection of cationic and neutral liposomes followed two minutes later by the HEDGES DNA vector, co-injection of dexamethasone covalently conjugated to the cationic liposomes together with large neutral liposomes, and species-specific optimized expression of CpG free DNA vectors [17]. In addition, we previously showed that HEDGES DNA-vectors neither detectably integrate into host genomic DNA nor elicit adaptive immune responses, thus enabling HEDGES to effectively and persistently produce re-dosed cDNA encoded proteins in immunocompetent mice. Furthermore, critical mouse toxicity markers remain at or near background levels following HEDGES administration [17].

We then created a 2^nd^-generation of hopefully significantly more powerful HEDGES DNA vectors that can significantly more durably produce cDNA-encoded proteins with naturally very short half-lives, such as hGLA and hGH following one HEDGES administration. Specifically, we created varieties of HEDGES-DNA vectors, each containing one of many different serum protein half-life extending DNA sequences. These protein half-life extending sequences included different Fc regions or serum albumin-derived sequences [19–22].

We then measured the duration of serum protein levels produced in outbred, immunocompetent CD-1 mice following one IV-administration of each of these DNA-vectors encoding either hGH or GLA. Finally, we measured the duration of hGLA serum protein levels produced following one IV-administration of our most efficient HEDGES hGLA DNA-vector into GLA knockout mice [23].

## Materials and methods

### Plasmid construction

The CpG-free gBlock fragments (Integrated DNA Technologies) containing R6K, Kan^r^, and multiple cloning sites (Dra III, Eco RI,and Nhe I) were assembled using the Gibson Assembly Method (NEB) as a 1.2-kb base vector. To accommodate multiple rounds of cloning into the base vector, the expression cassette was constructed in pUC19 by placing Eco RI–Nhe I and Xba I restriction sites at the 5′ and 3′ ends, respectively, flanking the expression cassette. The open reading frame (ORF) was inserted at Bst EII–Bgl II, and the enhancer/promoter was located between the Nhe I and Bst EII sites.

The polyadenylation site was between the Bgl II and Xba I sites. The Eco RI–Xba I expression cassette fragment was then inserted into the CpG-free base vector at the Eco RI–Nhe I sites. The cDNAs for hGLA, hGH and half-live extension sequence were ordered from GeneArt (Thermo Scientific) as codon optimized CpG-free sequence. The linker sequence consists of a short 5 amino acid sequence (Gly-Gly-Gly-Gly-Ser).

### DNA vector production

Plasmid was produced using Qiagen EndoFree Maxi kit (Qiagen: 12362) according to the manufacturer’s protocol. Plasmid was eluted with steriled lactated Ringer’s solution (LRS) and filter-sterilized with a 0.2-µm sterile syringe polyethersulfone membrane (Millipore).

### Liposome production

DOTAP and DMPC (14:0 PC 1,2-dimyristoyl-sn-glycero-3-phosphocholine) lipids were obtained from Avanti Polar Lipids (SKU 890890C and 850345C). Dexamethasone 21-palmitate (Dex-Palm) was obtained from Toronto Research Chemicals (D298830). MLV stocks were prepared by suspending DOTAP or DMPC, with or without Dex-Palm, in 5% dextrose in water.

### *In vivo* protocols and mice

Female outbred Hsd:ICR (CD-1®) mice (7 to 10 weeks old, from Envigo or Charles River Laboratories) were used for all studies. All mouse studies were conducted according to protocols approved by the Institutional Animal Care and Use Committee at the California Pacific Medical Center Research Institute. The health of aminals is closely monitored throughout the experiment. At the end point, mice were euthanized by CO2 narcotization, followed by either bilateral thoracotomy with cardiac transection.

### HEDGES IV injections

The most technically challenging aspect of HEDGES IV administration is the sequential injection itself which is described here in more detail: Mice were given intraperitoneal injections of water-soluble dexamethasone (40mg/kg) in Lactated Ringers solution. Two hours later, mice are placed for 2-3 minutes under a warming lamp to slightly dilate the tail veins, watched closely for any signs of heat-related distress.

Mice are placed into an injection restrainer and the tail swabbed with rubbing alcohol. A 28g/29g insulin syringe is inserted at a shallow angle into the distal half of one of the lateral tail veins and the solution of 1000 nmol DOTAP (1,2-dioleoyl-3-trimethylammonium-propane) liposomes containing 2.5% w/v dexamethasone 21-palmitate and 1000nmol DMPC (1,2-dimyristoyl-sn-glycero-3-phosphocholine) liposomes containing 5% w/v dexamethasone 21-palmitate, in Lactated Ringers is injected quickly into the vein. When the needle is withdrawn the puncture on the tail is held closed for 30-45 seconds until a clot has formed and bleeding stops.

Two minutes after the initial injection, a second injection containing the DNA plasmid is given, either into the same tail vein more proximal to the mouse body, or into the vein on the opposite side, in the same manner as before. The first puncture is held closed during the second injection to ensure the clot is not disturbed. The second puncture is held closed for the same length of time before the mouse is returned to the cage and monitored during recovery. Any animal display respiratory difficulty, lethargy or restricted mobility will be euthanized immediately as previously described.

### Blood collection

Mice were anesthetized with isoflurane and bled via submandibular puncture into serum separator tubes. After the sample has been collected, pressure will immediately be applied with a gauze sponge until bleeding has stopped. The animal will then immediately be returned to a warm cage and monitored for return of a normal respiratory pattern and movement until fully conscious. General appearance and signs of active bleeding or hematoma at collection site will be monitored several times in the first 30-60 minutes after blood is collected, and then 1 day after each collection. Once the animal has recovered fully from the anesthesia, it will be returned to the home cage.

### Tissue collection and processing

Mice were anesthetized to the surgical plane, and the chest cavity opened. A needle was inserted into the ventricle and mice were perfused with 10mL sterile PBS until lungs were white. Organs were harvested and washed in PBS on ice, then homogenized with tissue homogenizer in lysis buffer on ice. Total protein was quantified by BCA assay from Pierce.

### Protein concentration by ELISA

A specific ELISA for hGLA from Raybiotech (PO6280 ELH-aGLA) was used for quantitation of hGLA protein level in serum according to the protocol. The hGH ELISA was coated with sheep anti-hGH capture antibody (Abcam: ab64499) and detected with rat anti-GH, HRP detection antibody (Abcam: ab06749) using recombinant hGH (Abcam: 116162) as a protein standard. Statistics were calculated using Student’s t-test.

#### Bioactivity assay.

A fluorometric activity assay (Abcam ab239716) was used for quantification of GLA bioactivity in serum, according to manufacture’s protocol.

## Results

### A novel HEDGES hGLA DNA vector

**Fig 1:**
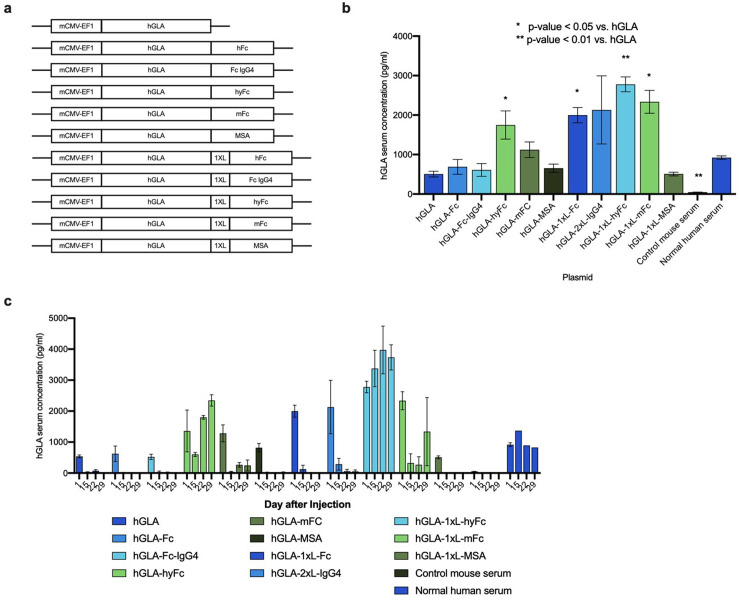
Expression of various hGLA plasmid DNA vectors. **a**, Schematic of each of the hGLA plasmids injected. hGLA: 1300 bp, hFc: 750 bp, Fc IgG4: 750 bp, hyFc: 750 bp, mFc: 750 bp, MSA: 1800, 1XL: 15 bp. **b**, hGLA ELISA data from mouse serum 24 hours post treatment. Data is presented as mean ±  SEM (n = 5). **c**, hGLA ELISA data from mouse serum collected at days 1, 15, 22, and 29. Data is presented as mean ±  SEM (n = 5).

Current ERT treatments for Fabry disease require frequent re-injections due to the short (<20 minute) half-life of endogenous hGLA protein [5]. To attempt to significantly extend the half-life of the HEDGES DNA vector cDNA-encoded hGLA protein, we constructed a series of different HEDGES DNA vectors, each vector encoding the codon-optimized human hGLA wild type cDNA, with or without the addition of one of 5 distinct but similar half-life extending fragments at the 3’ end (Fig 1a. Specifically, these fragments included a human fragment crystallizable region (Fc) of human IgG1, mouse Fc (mFc) of mouse IgG2a, hybrid Fc (hyFc), mouse serum albumin (MSA), or Fc of human IgG4 [19,20,22].

In addition, HEDGES individual DNA vectors were constructed containing one or two copies (1xL or 2xL) of a 5 amino acid linker inserted between hGLA and each half-life extending molecule. Flexible linkers are short chains of small polar amino acids (in this case the 5 residue linker GGGGS) can provide improvement in protein folding and stability, facilitate protein expression, preserve or improve biological function, and/or alter the pharmacokinetic stability, facilitate protein expression, preserve or improve biological function, and/or alter the pharmacokinetic profile [21]. These HEDGES DNA vectors were then injected IV into immunocompetent CD-1 mice.

Specifically, cationic and neutral liposomes were injected IV followed two minutes later by plasmid DNA as previously described [17]. Serum from each injected mouse was collected after 24 hours and analyzed for hGLA serum levels using a human GLA-specific ELISA assay. A control group of un-injected, ~  25 gm CD-1 female mice obtained from either Charles River labs or Envigo labs were used together with all the test groups in every experiment presented in this manuscript. In every experiment performed, values obtained from control mice did not statistically significantly differ from assay background levels.

The results show that 4 of the 10 different HEDGES DNA vectors containing protein half-life extending DNA sequences produced significantly higher serum hGLA serum levels when compared to the HEDGES hGLA DNA vector that lacked a protein half-life extender DNA sequence (Fig 1b). Unexpectedly, the hGLA-1xL-hyFc HEDGES DNA vector produced substantially the highest hGLA serum levels of all the similar HEDGES GLA DNA vectors tested. Specifically, when compared to the hGLA-hyFc HEDGES DNA vector, the addition of five amino acids linker, substantially increased hGLA serum protein levels over time. Conversely, MSA did not significantly increase hGLA serum levels regardless of the presence or absence of a linker region.

To assess the longer-term effects on hGLA serum levels produced by each of the different HEDGES hGLA DNA vector variations shown in Fig 1b, we serially analyzed hGLA serum levels produced over time post-injection. Despite higher serum hGLA serum levels produced 24 hours post-injection by 4 of the different HEDGES hGLA DNA vectors injected, only the HEDGES hGLA-hyFc-1xL DNA vector produced hGLA serum levels in the 1,000–5,000 pg ml range at day 15 post-injection (Fig 1c). Mice treated with the hGLA HEDGES DNA vector lacking any half-life extender sequence showed approaching baseline hGLA serum levels after the day 1 timepoint. Additionally, the mFc-containing HEDGES DNA vectors, despite producing serum hGLA levels in the 1,000 pg/ml range at day 1, did not produce significantly elevated hGLA serum levels thereafter.

### HEDGES Re-adminstration

**Fig 2:**
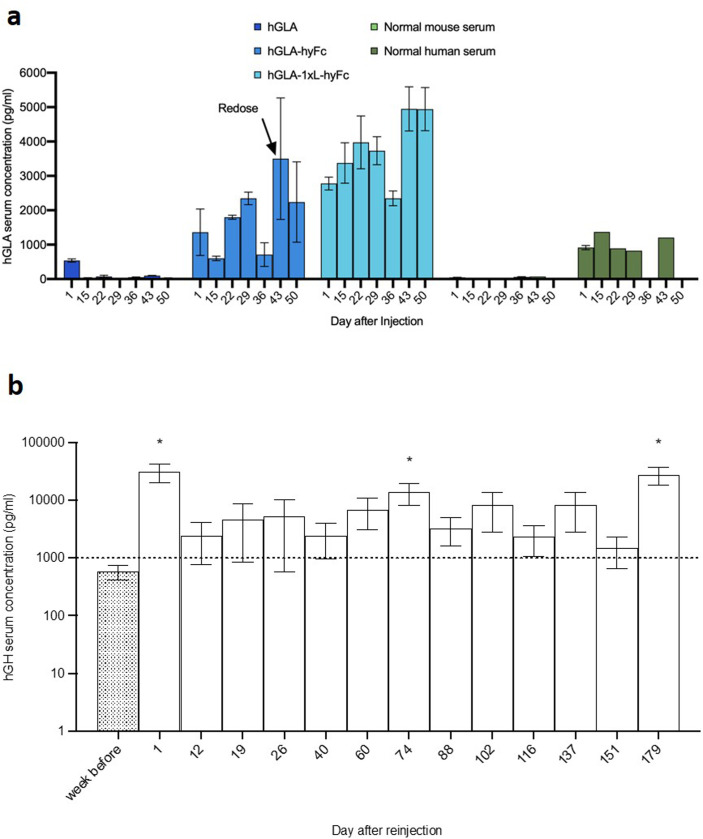
Extended expression of hGLA and hGH with re-injection. **a)** Mice were injected on day 0 with the hGLA construct as shown. The three mice injected with hGLA-hyFc were re-injected on day 42 with the same construct. Serum was collected and analyzed by ELISA every 7 or 14 days. Data is presented as mean ±  SEM (n = 3). **b)** Mice were previously injected IV with an hGH HEDGES DNA-vector. The value before reinjection is indicated as week before. Mice were reinjected intravenously with an hGH HEDGES and serum was collected and analyzed by ELISA every 1 to 2 weeks. Data is presented as mean ±  SEM (n = 7). *  p-value <  0.05.

Previously, re-injection of a HEDGES DNA vector encoding the hG-CSF cDNA in immunocompetent CD-1 mice significantly increased the already elevated hG-CSF levels for a prolonged period with no adverse events [17]. Therefore, to attempt to increase hGLA serum levels produced by the HEDGES hGLA-hyFc DNA vector, it was re-injected 42 days after initial injection in a group of three mice. One HEDGES hGLA-hyFc DNA vector re-injection further increased serum hGLA levels at days 43 and 50 post injection (Fig 2a). Similar to hGLA, we also show that re-injection of hGH HEDGES DNA-vector increased the hGH serum and maintained above 1,000 ng/ml up to 179 days post re-injection (Fig 2b).

### Modification of the DNA vector Fc region

Unlike the other similar HEDGES hGLA DNA vectors tested, the HEDGES hGLA-1xL-hyFc DNA vector maintained hGLA serum levels approaching 10,000 pg/ml for more than 36 days (Fig 2). In attempt to further extend the half-life of hGLA, we performed either single or combinatorial site-directed base mutations on the Fc fragment to increase binding affinity to the neonatal Fc receptor (FcRn) (Hinton et al., 2004). The avidity of the interaction of Fc-FcRn at the lower pH (6.0–6.5) in endosomes allows the Fc molecule to escape lysosomal degradation and recycles it to the blood stream and interstitial tissue space, which is at a higher pH (7.0–7.5). This has been shown to contribute to Fc half-life extension [21]. We then determined if single or combination mutations could significantly alter hGLA serum half-life.

**Fig 3:**
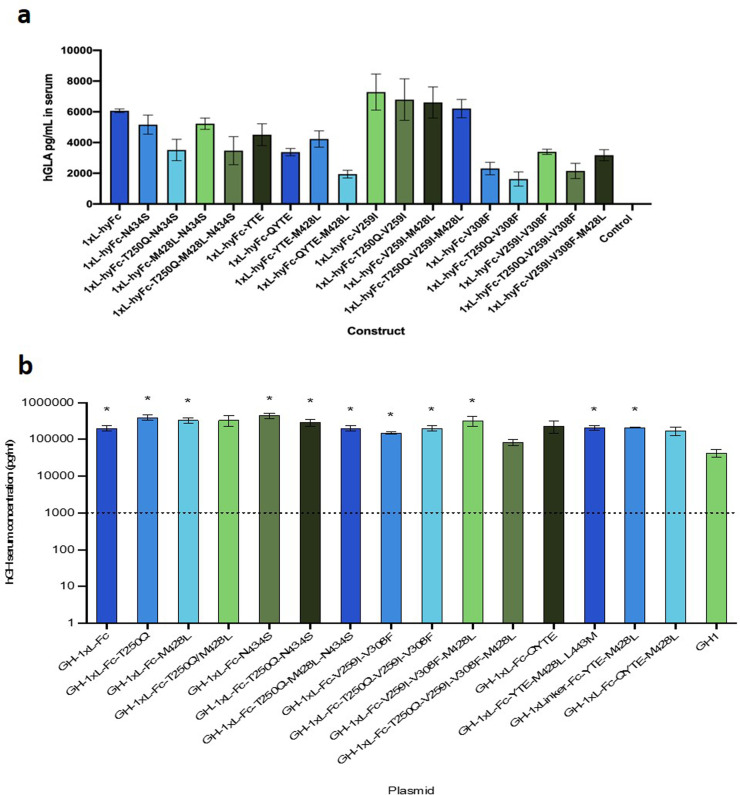
Fc point mutations do not enhance expression. **a)** hGLA serum levels following IV HEDGES injection. Mice were injected with constructs shown and serum collected 24 hours later and analyzed by ELISA. Data is presented as mean ±  SEM (n = 3). **b)** hGH serum expression levels following IV HEDGES injection. Mice were injected with constructs shown and serum collected 24 hours later and analyzed by ELISA. Data is presented as mean ±  SEM (n = 3). *  p-value <  0.05.

Specifically, groups of mice were injected with either the previous HEDGES hGLA-1xL-hyFc DNA vector or one of 17 mutated HEDGES hGLA-1xL-hyFc DNA vectors [20]. None of the base modifications tested significantly increased hGLA serum levels when compared to the unmodified hGLA-1xL-hyFc HEDGES DNA vector (Fig 3a). Thus, a 15-base linker produces substantially higher, more durable hGLA serum levels than the HEDGES hGLA-hyFc DNA vector, which differs by only 15 bases from the hGLA-1xL-hyFc HEDGES DNA vector.

### Elevated hGLA levels in heart tissue

**Fig 4:**
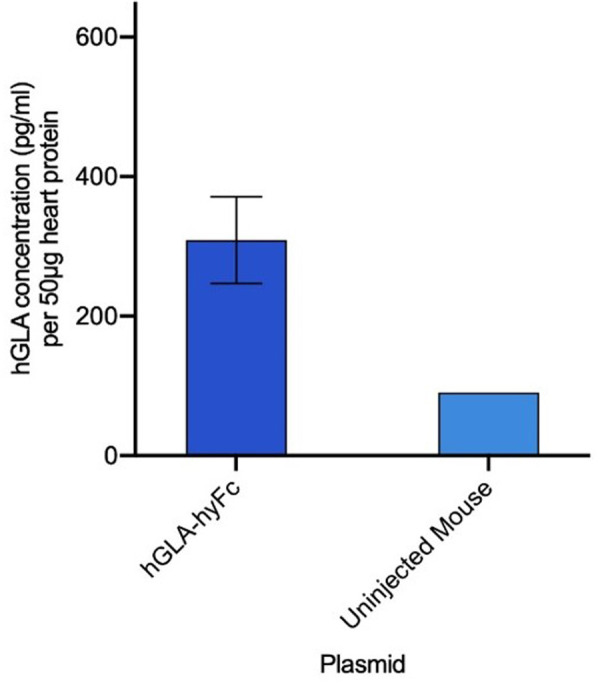
hGLA expression in heart tissue. hGLA ELISA data from heart tissue extracted from IV HEDGES treated mice on day 104. Data is presented as mean ±  SEM (n = 3).

Since GLA is a lipid processing enzyme active endogenously within tissues, we measured the level of hGLA in the heart, an organ often severely affected in Fabry patients [3]. Specifically, CD-1 mice which had either earlier received one injection of the HEDGES hGLA-1xL-hyFc DNA vector or remained un-injected were terminally bled 104 days later. Hearts were collected and processed, and samples run on the hGLA-specific ELISA. The hearts from injected mice expressed significantly higher levels of hGLA protein than un-injected controls (Fig 4).

### Long-term levels of hGLA in the 1,000 to 10,000 pg/ml range

**Fig 5:**
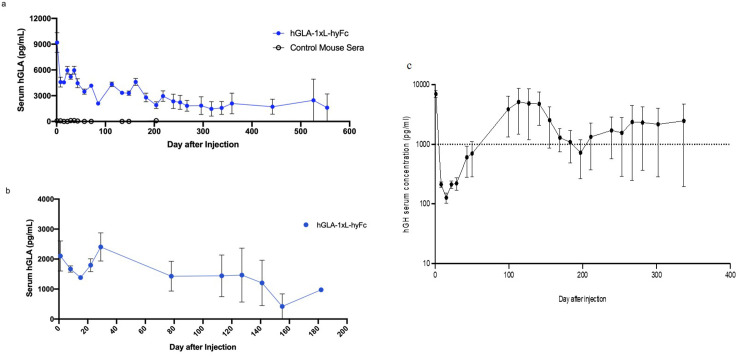
Long-term expression of hGLA or hGH in wild-type and knockout mice (hGLA). **a)** Expression of hGLA over time in CD-1 mice by ELISA. Mice were **IV** HEDGES injected with hGLA-1xL-hyFc, and serum collected and analyzed every 1, 2, or 3 weeks. Data is mean ± SEM (n = 4). **b)** hGLA in GLA knockout mice. hGLA ELISA data for KO mice after **IV** HEDGES treatment. Samples were collected every 1, 2, 3, or 4 weeks. Data is presented as mean ±  SEM (n = 4). **c)** Serum expression levels of hGH over time in CD-1 mice by ELISA. Mice were **IV** HEDGES injected with hGH and serum was collected and analyzed every 1 to 2 weeks (there was a 50 day gap between days 50 and 100). Data is mean ± SEM (n = 4). Horizontal hatched line at 1 ng per ml in Fig 5c indicates that 1 ng per ml is the lower end of hGH serum levels. hGLA serum levels produced at every time point in the HEDGES hGLA DNA vector-injected group are statistically significantly higher (p <  0.05) and those produced in the control mice. All hGH serum levels in control mice remained at background levels throughout the experiment.

HEDGES-based single dose administration of the HEDGES hGLA-1xL-hyFc DNA vector was first tested in immunocompetent, outbred CD-1 mice to determine how long it produced serum hGLA protein levels in the 1,000 to 5,000 pg/ml range. One HEDGES hGLA DNA vector injection into CD-1 mice produced serum hGLA levels for greater than the next 550 mouse days in the 1,000 to 10,000 pg/ml range, thus increasing the duration of hGLA serum protein levels produced by > 38,100 fold versus wildtype-protein (Fig 5a). One intravenous-administration of this same 2^nd^-generation HEDGES GLA DNA-vector produced serum GLA levels for > 160 days in the 1,000 to 10,000 pg/ml range in GLA knockout-mice, a 2,800 fold increase versus wildtype protein (Fig 5b). Of note, outbred CD1 mice produced higher mean serum hGLA levels than did immunocompetent GLA-null knockout mice. Whereas outbred CD1 mice produce normal levels of mouse GLA, GLA-null knockout mice produce no endogenous GLA protein of any kind. Human GLA protein shares significant homology to mouse GLA protein. Therefore, CD-1 mouse immune responses directed against human GLA protein should be significantly less intense than immune responses in immunocompetent GLA-null knockout mice. This results in more durable hGLA serum protein levels observed in outbred CD1 mice. Of interest, the 1^st^-generation HEDGES DNA vector that dramatically increased the duration of hG-CSF-protein levels in the 1,000 to 10,000 pgml range produced in CD-1 mice [17]. The 1^st^-generation HEDGES DNA vector also increased the duration of hGH serum levels in the 1,000 to 10,000 pg/ml range produced in CD-1 mice by 22,860 fold when compared to the duration of hGH serum levels produced by hGH recombinant wildtype hGH protein. (Fig 5c). Conversely, the 1^st^-generation HEDGES hGLA DNA vector did not increase the duration of hGH serum levels produced in CD-1 mice when compared to recombinant hGLA protein. (Fig 5c). Only the 2^nd^-generation HEDGES hGLA DNA vector substantially increased duration of hGLA serum levels produced in the 1,000 to 10,000 pg/ml range in CD-1 mice(Fig 1b and 1c).

### Full bioactivity of HEDGES-driven hGLA

**Fig 6:**
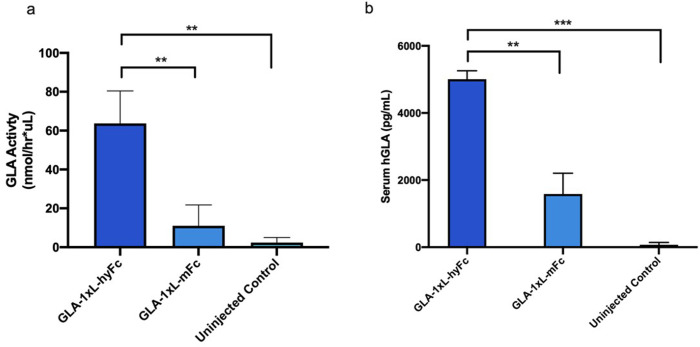
Correlation of hGLA enzyme activity assay and expression level. Mice were HEDGES-injected with hGLA-1xL-hyFc or hGLA-1xL-mFc, or uninjected. **a**, hGLA activity assay levels, by fluorometric activity assay. Data is mean ±  SEM (n = 3). **b**, hGLA ELISA assay levels from the same serum samples. Data is mean ±  SEM (n = 3). ** p-value < .01; *** p value < .001.

To verify that the hGLA protein expressed in HEDGES-injected mice is bioactive, the enzymatic activity of HEDGES-driven hGLA was measured by a fluorometric activity assay. This approach allowed us to directly compare results as measured by an hGLA bioassay to those measured by an ELISA immunoassay. Using serum collected from HEDGES-injected or un-injected control mice, both enzymatic activity quantification (Fig 6a) and immunoreactive protein quantification (Fig 6b) were performed on day 57 post-injection. The hGLA serum enzymatic activity correlated closely with the level of hGLA protein detected in serum.

## Discussion

Fabry Disease (FD), Growth Hormone deficiency (GHD) and hG-CSF deficiency are three examples of rare human monogenic deficiency diseases, each caused by a single protein deficiency. FD is progressive, highly symptomatic, compromises many different organs, and is ultimately fatal if untreated[3,5]. GHD can be congenital, acquired or idiopathic. Congenital GHD delays or deforms physical maturation. While ERT is an effective therapy, its high cost and frequent intravenous administration each pose significant limitations. The very short serum protein half-lives of hGLA and hGH limit current ERT therapeutic interventions for both FD and GHD. Specifically, hGLA ERT requires bi-weekly infusions for the life of the patient, costs ~ $300,000 per-patient per-year, is not effective in all patients, and is essentially never curative[3,5,7].

In addition, individuals with Fabry disease who cannot access ERT may die prematurely from accelerated disease progression. Furthermore, ERT administration is often associated with infusion reactions that can elicit life-threatening anaphylactic reactions in some individuals, rendering them unable to continue therapy[3,5].HGH ERT requires even more frequent administration than hGLA ERT with up to daily administration[7].

Therefore, a safer, more cost effective, single-dose platform that effectively and durably functionally replaces now absent monogenic deficiency proteins is urgently needed. We previously showed that one IV-administration of our 1^st^-generation HEDGES hG-CSF DNA vector into outbred, immunocompetent CD-1 mice produced critical therapeutic endpoints for > 582 days. Specifically, it produced hG-CSF’s two critical clinical therapeutic endpoints: significantly elevating circulating absolute neutrophil counts as well as partially replacing normal haematopoietic precursor cells with immature neutrophil precursor cells in both bone marrow and spleen[17]. Therefore, HEDGES-based administration increased the duration of hG-CSF serum protein levels produced by > 13,900 fold versus administering wildtype recombinant hG-CSF protein therapy.

Here we demonstrate that one IV-adminstration of our 1^st^-generation HEDGES DNA -vector encoding the wildtype hGH protein produced hGH serum levels in the 1,000 to 10,000 pg/ml range for > 337 days, thus increasing the duration of hGH serum protein levels produced by >  24,200 fold compared to administering wildtype, recombinant hGH protein therapy. Conversely, our 1^st^-generation of HEDGES hGLA DNA vector produced hGLA seurm protein levels less than 1,000 pg/ml for less than 14 days in identical CD-1 mice. In an attempt to significantly increase the duration of short half-life proteins, 2^nd^ generation HEDGES DNA vectors were constructed. Our HEDGES hGLA 1x hyFc DNA vector produced hGLA serum levels in the 1,000 to 10,000 pg/ml range for more than 550 days in outbred, incompetent CD-1 mice. Thus, HEDGES-based administration increased the duration of hGLA serum protein levels produced by >  38,100 fold vesus administering recombinant, wildtype hGLA protein therapy (Fig 5b). Furthermore, the extended expression of HEDGES hGLA 1xhyFc for more than 160 days represents a 2.800 fold over administering wildtype, recombinant hGLA protein therapy in GLA knockout-mice (Fig 5c). Since mouse and human GLA proteins are highly homologous, outbred mice would produce a less intense immunological response to the foreign GLA protein than those produced by GLA-null knockout mice. This explains the higher serum hGLA levels produced by the 2^nd^-generation HEDGES hGLA DNA vector in outbred CD-1 mice versus in genetically modified GLA-null knockout mice [Table 1].

**Table 1:** HEDGES versus BPPTs versus AAV.

Critical parameter	HEDGES	BRPTs	AAV
Duration of therapeutic serum protein levels produced post one administration	Durable [17]	One day to 3 weeks [2]	Durable[10]
Can be re-dosed	Yes [17]	Yes [2]	No [12]
Causes life-threatening allergic infusion reactions	No [17]	Yes [5]	No [12]
Can elicit fatal adaptive immune responses	No [17]	No [5]	Yes [12]
Produces durable therapeutic levels of range of FDA-approved protein therapies	Yes [17]	No [5]	Yes [10]
Produced *in vivo or ex vivo*	*In vivo* [17]	*Ex vivo* [5]	*In vivo* [10]
Integrates into genomic DNA	No [17]	No [5]	Yes [13]
Requires an intact cold chain	No [17]	Yes [5]	Yes [10]
Can be freeze dried	Yes [17]	No [5]	Yes [24]

* Bioreactor-produced Recombinant Protein Therapies (BRPTs).

Until now, most nonviral, DNA vector-based technologies have remained unable to produce durable therapeutic serum levels of biologically and therapeutically important human proteins in immunocompetent animals [24]. Here, only one of the 11 similar HEDGES hGLA DNA vectors (the HEDGES hGLA-1xL-hyFc DNA vector), administered to identical groups of immunocompetent CD-1 mice, produced hGLA serum levels in the 1,000 to 10,000 pg/ml range for > 550 days following one HEDGES DNA vector injection (Fig 1b). Specifically, when compared to the hGLA-hyFc HEDGES DNA vector, the addition of a single 15 base linker region (1xL) between hGLA and hyFc, (hGLA-1xL-hyFc) enabled the HEDGES hGLA-1xL-hyFc DNA vector to produce serum hGLA levels in the 1,000 to 10,000 pg/ml range for ≥  550 days. Therefore, a difference of only 15 bases, five amino acids, dramatically increases hGLA serum levels produced over time following one IV administration.

Unlike the substantial hGLA serum protein half-life prolongation produced by the 1xL-hyFc hGLA DNA vector configuration documented in Fig 2b, the 1xL-hyFc hGLA DNA vector configuration does not extend the hGH serum protein half-life when compared to each of the 10 other 2^nd^-generation HEDGES hGH DNA vectors that each contain a different serum protein half-life extender DNA sequence (Fig 2c). Many different factors appear to have enabled this singular ability of the 1xL-hyFc hGLA DNA vector configuration to drammatically extend hGLA serum protein half-life. These include, first, hyFc is a combination of the IgD hinge region and the CH2 and CH3 regions of human IgG4 [25,26].

Specifically, most human Fc fragments used to extend serum protein half-lives are contributed by the human IgG1 Fc region. IgG1 Fc binds to the FcγI receptor as well as to the complement component 1q (C1q) region, which leads to antibody-dependent cellular cytotoxicity (ADCC) and/or complement-dependent cytotoxicity (CDC) [25,26].

ADCC as well as CDC would progressively reduce the number HEDGES DNA vector transfected cells in the body, thus progressively reducing hGLA serum levels over time, as demonstrated in Fig 2A. Conversely, human IgG4 Fc does not bind C1q and binds FcγI only at low levels. In addition, the hinge region of IgG4 is less flexible than IgG1, which could result in steric hindrance between the bioactive molecule (hGLA) and the half-life extending molecule.

In our construct, the hinge region of hIgG4 is replaced with a segment of the most flexible hinge region of hIgD. In addition, the HEDGES hGLA-1xL-HyFc DNA vector contains the 1xL linker, further improving the expressed protein’s flexibility and folding by separating the GLA domain from the IgG4 Fc domain and resulting in an increased functional GLA yield over time. We also attempted to further enhance expression of our hGLA-1xL-hyFc and hGH-1xL-hyFc by altering Fc affinity to FcRn. There are several reports successfully increasinghalf-life of target protein by Fc mutations[21,22,27].

However, in this study, those previously reported mutations either alone or in combination did not increase expression of hGLA-1xL-hyFc (Fig 3A). Interestingly, the hGH serum protein expression levels are not enhanced by the presence of the hyFc protein half-life extender sequence with or without the linker. This suggests that the modification of half-life extending sequences is gene-specific. The successful application of one particular Fc region sequence to extend the half-life of one protein is not a guaranteed success in another protein. This was confirmed when the same Fc region point mutations in the hGLA and hGH HEDGES DNA vectors yielded very different durations following one intravenous administration (Fig 3B).

Another advantage of the HEDGES DNA-vector technology is its ability to efficiently, durably and re-express HEDGES DNA vector cDNA-encoded genes following one re-dose in immunocompetent mice. Extended production of hGLA serum protein levels were produced when the identical HEDGES DNA-vector was re-injected once into the same immunocompetent mice. To date, we have not tested more than one HEDGES re-dose in mice.

Despite ERT being the standard-of-care for many human monogenic protein deficiency diseases, a major consideration is that all recombinant proteins, including ERT, are now produced in costly bioreactors [28]. In contrast, HEDGES obviates the need for bioreactor generated recombinant protein production by continuously producing recombinant proteins within, then secreting these proteins from, hundreds of millions of HEDGES DNA vector transfected vascular endothelial cells in the host [17]. Furthermore, unlike ERT, neither of the two components of HEDGES, plasmid DNA vectors and liposomes, requires an intact cold chain [29]. Rather, each is readily freeze dried, enabling HEDGES to be stored for prolonged periods at ambient temperatures, even in equatorial regions [29]. HEDGES’ ability to much more fully access under-resourced areas worldwide may be critical to progressively treating these many thousands of rare human monogenic deficiency diseases in these severely under resourced areas worldwide.

Last, we exclusively used immunocompetent CD-1 or GLA KO mice in these studies, together with HEDGES DNA vectors that encode fully human GLA or hGH proteins. As shown previously, one IV injection of a HEDGES DNA vector encoding rituximab, a largely humanized, FDA approved mAb, elicits highly neutralizing, mouse anti-human protein antibody responses in up to one third of injected mice over time [17]. The onset of highly neutralizing mouse anti-human antibody responses produces an interspecies artifact that then rapidly returns rituximab serum protein levels to background levels. Therefore, HEDGES DNA vector-based delivery of cDNA encoded human proteins should prove even more effective in humans than in immunocompetent mice.

Taken together, the HEDGES-based results presented here may enable one safe intravenous administration of either a first- or second-generation HEDGES DNA vector to durably as well as safely functionally correct one or more of these now many thousands of now incurable, rare human monogenic deficiency diseases deficiency diseases. Importantly, one administration of our 2^nd^-generation HEDGES hGLA-hyFc-1xL DNA vector produces durable hGLA serum levels in the 1,000 to 10,000 pg/ml in GLA knockout-mice. Previously, major therapeutic responses produced by GLA ERT, as well as by other novel anti-Fabry disease therapies in GLA knockout-mice have accurately-predicted subsequent major therapeutic responses in Fabry patients [30,31]. Therefore,we will attempt to initiate IND-enabling large animal studies as a prelude to follow-on phase 1 human clinical trials in Fabry patients.

## References

[1] Wang Y, Hu LF, Zhou TJ, Qi LY, Xing L, Lee J, et al. Gene therapy strategies for rare monogenic disorders with nuclear or mitochondrial gene mutations. Biomaterials. 2021;277:121108. doi: 10.1016/j.biomaterials.2021.121108 34478929

[2] Condò I. Rare monogenic diseases: molecular pathophysiology and novel therapies. Int J Mol Sci. 2022;23(12):6525. doi: 10.3390/ijms23126525 35742964 PMC9223693

[3] Ioannou YA, Zeidner KM, Gordon RE, Desnick RJ. Fabry disease: preclinical studies demonstrate the effectiveness of alpha-galactosidase A replacement in enzyme-deficient mice. Am J Hum Genet. 2001;68(1):14–25. doi: 10.1086/316953 11115376 PMC1234907

[4] Solomon M, Muro S. Lysosomal enzyme replacement therapies: historical development, clinical outcomes, and future perspectives. Adv Drug Deliv Rev. 2017;118:109–34. doi: 10.1016/j.addr.2017.05.004 28502768 PMC5828774

[5] Germain DP. Fabry disease. Orphanet J Rare Dis. 2010;5:30. doi: 10.1186/1750-1172-5-30 21092187 PMC3009617

[6] Germain DP, Charrow J, Desnick RJ, Guffon N, Kempf J, Lachmann RH, et al. Ten-year outcome of enzyme replacement therapy with agalsidase beta in patients with Fabry disease. J Med Genet. 2015;52(5):353–8. doi: 10.1136/jmedgenet-2014-102797 25795794 PMC4413801

[7] Shalet SM, Toogood A, Rahim A, Brennan BM. The diagnosis of growth hormone deficiency in children and adults. Endocr Rev. 1998;19(2):203–23. doi: 10.1210/edrv.19.2.0329 9570037

[8] Cai Y, Xu M, Yuan M, Liu Z, Yuan W. Developments in human growth hormone preparations: sustained-release, prolonged half-life, novel injection devices, and alternative delivery routes. Int J Nanomedicine. 2014;9:3527–38. doi: 10.2147/IJN.S63507 25114523 PMC4122423

[9] Mameli C, Orso M, Calcaterra V, Wasniewska MG, Aversa T, Granato S, et al. Efficacy, safety, quality of life, adherence and cost-effectiveness of long-acting growth hormone replacement therapy compared to daily growth hormone in children with growth hormone deficiency: A systematic review and meta-analysis. Pharmacol Res. 2023;193:106805. doi: 10.1016/j.phrs.2023.106805 37236413

[10] Chu WS, Ng J. Immunomodulation in administration of rAAV: preclinical and clinical adjuvant pharmacotherapies. Front Immunol. 2021;12:658038. doi: 10.3389/fimmu.2021.658038 33868303 PMC8049138

[11] High-dose AAV gene therapy deaths. Nat Biotechnol. 2020;38(8):910. doi: 10.1038/s41587-020-0642-9 32760031

[12] Weber T. Anti-AAV antibodies in AAV gene therapy: current challenges and possible solutions. Front Immunol. 2021;12:658399. doi: 10.3389/fimmu.2021.658399 33815421 PMC8010240

[13] Nguyen GN, Everett JK, Kafle S, Roche AM, Raymond HE, Leiby J, et al. A long-term study of AAV gene therapy in dogs with hemophilia A identifies clonal expansions of transduced liver cells. Nat Biotechnol. 2021;39(1):47–55. doi: 10.1038/s41587-020-0741-7 33199875 PMC7855056

[14] Nowak A, Mechtler TP, Desnick RJ, Kasper DC. Plasma LysoGb3: a useful biomarker for the diagnosis and treatment of Fabry disease heterozygotes. Mol Genet Metab. 2017;120(1–2):57–61. doi: 10.1016/j.ymgme.2016.10.006 27773586

[15] Askari H, Kaneski CR, Semino-Mora C, Desai P, Ang A, Kleiner DE, et al. Cellular and tissue localization of globotriaosylceramide in Fabry disease. Virchows Arch. 2007;451(4):823–34. doi: 10.1007/s00428-007-0468-6 17674039

[16] Valbuena C, Leitão D, Carneiro F, Oliveira JP. Immunohistochemical diagnosis of Fabry nephropathy and localisation of globotriaosylceramide deposits in paraffin-embedded kidney tissue sections. Virchows Arch. 2012;460(2):211–21. doi: 10.1007/s00428-011-1182-y 22205110

[17] Handumrongkul C, Ye AL, Chmura SA, Soroceanu L, Mack M, Ice RJ, et al. Durable multitransgene expression in vivo using systemic, nonviral DNA delivery. Sci Adv. 2019;5(11):eaax0217. doi: 10.1126/sciadv.aax0217 31807699 PMC6881169

[18] Heussner P, Haase D, Kanz L, Fonatsch C, Welte K, Freund M. G-CSF in the long-term treatment of cyclic neutropenia and chronic idiopathic neutropenia in adult patients. Int J Hematol. 1995;62(4):225–34. 8589368

[19] Strohl WR. Fusion proteins for half-life extension of biologics as a strategy to make biobetters. BioDrugs. 2015;29(4):215–39. doi: 10.1007/s40259-015-0133-6 26177629 PMC4562006

[20] Monnet C, Jorieux S, Urbain R, Fournier N, Bouayadi K, De Romeuf C, et al. Selection of IgG variants with increased FcRn binding using random and directed mutagenesis: impact on effector functions. Front Immunol. 2015;6:39. doi: 10.3389/fimmu.2015.00039 25699055 PMC4316771

[21] Chen X, Zaro JL, Shen WC. Fusion protein linkers: property, design and functionality. Adv Drug Deliv Rev. 2013;65(10):1357–69. doi: 10.1016/j.addr.2012.09.039 23026637 PMC3726540

[22] Hinton PR, Johlfs MG, Xiong JM, Hanestad K, Ong KC, Bullock C, et al. Engineered human IgG antibodies with longer serum half-lives in primates. J Biol Chem. 2004;279(8):6213–6. doi: 10.1074/jbc.C300470200 14699147

[23] Ohshima T, Murray GJ, Swaim WD, Longenecker G, Quirk JM, Cardarelli CO, et al. alpha-galactosidase A deficient mice: a model of Fabry disease. Proc Natl Acad Sci U S A. 1997;94(6):2540–4. doi: 10.1073/pnas.94.6.2540 9122231 PMC20124

[24] Zu H, Gao D. Non-viral vectors in gene therapy: recent development, challenges, and prospects. AAPS J. 2021;23(4):78. doi: 10.1208/s12248-021-00608-7 34076797 PMC8171234

[25] Robbie GJ, Criste R, Dall’acqua WF, Jensen K, Patel NK, Losonsky GA, et al. A novel investigational Fc-modified humanized monoclonal antibody, motavizumab-YTE, has an extended half-life in healthy adults. Antimicrob Agents Chemother. 2013;57(12):6147–53. doi: 10.1128/AAC.01285-13 24080653 PMC3837853

[26] Zalevsky J, Chamberlain AK, Horton HM, Karki S, Leung IWL, Sproule TJ, et al. Enhanced antibody half-life improves in vivo activity. Nat Biotechnol. 2010;28(2):157–9. doi: 10.1038/nbt.1601 20081867 PMC2855492

[27] Sockolosky JT, Szoka FC. The neonatal Fc receptor, FcRn, as a target for drug delivery and therapy. Adv Drug Deliv Rev. 2015;91:109–24. doi: 10.1016/j.addr.2015.02.005 25703189 PMC4544678

[28] Kelley B. Industrialization of mAb production technology: the bioprocessing industry at a crossroads. MAbs. 2009;1(5):443–52. doi: 10.4161/mabs.1.5.9448 20065641 PMC2759494

[29] Ando S, Putnam D, Pack DW, Langer R. PLGA microspheres containing plasmid DNA: preservation of supercoiled DNA via cryopreparation and carbohydrate stabilization. J Pharm Sci. 1999;88(1):126–30. doi: 10.1021/js9801687 9874713

[30] Nguyen Dinh Cat A, Escoubet B, Agrapart V, Griol-Charhbili V, Schoeb T, Feng W, et al. Cardiomyopathy and response to enzyme replacement therapy in a male mouse model for Fabry disease. PLoS One. 2012;7(5):e33743. doi: 10.1371/journal.pone.0033743 22574107 PMC3344819

[31] Zhu X, Yin L, Theisen M, Zhuo J, Siddiqui S, Levy B, et al. Systemic mRNA therapy for the treatment of Fabry disease: preclinical studies in wild-type mice, fabry mouse model, and wild-type non-human primates. Am J Hum Genet. 2019;104(4):625–37. doi: 10.1016/j.ajhg.2019.02.003 30879639 PMC6451694

